# Osteopontin in the Central Nervous System: Roles in Development, Injury, Neurodegeneration, and Neuro-Oncology

**DOI:** 10.3390/biom16070996

**Published:** 2026-07-07

**Authors:** Wei Zhang, Xianji Wei, Minyou Chen, Lingli Zhang, Jun Zou

**Affiliations:** 1 School of Exercise and Health, Shanghai University of Sport, Shanghai 200438, China; 2 College of Athletic Performance, Shanghai University of Sport, Shanghai 200438, China

**Keywords:** secreted phosphoprotein 1, osteopontin, central nervous system, microglia, neuroinflammation, glioblastoma

## Abstract

Osteopontin (OPN), encoded by the SPP1/Spp1 gene, is increasingly recognized as an extracellular matrix-associated immunoregulatory molecule in the central nervous system (CNS). In CNS-related contexts, OPN does not act as a uniformly protective or detrimental factor. Instead, its effects depend on the producing cell type, molecular form, receptor axis, disease stage, and lesion compartment. Accumulating evidence indicates that OPN may participate in reparative processes, including tissue preservation, debris clearance, vascular remodeling, and support of myelin-related repair, while sustained or ectopic OPN activity may contribute to synaptic injury, persistent glial reactivity, remyelination failure, and immunosuppressive tumor progression. In this review, we summarize the molecular basis of SPP1/Spp1 expression and OPN protein signaling, with emphasis on isoforms, proteolytic processing, receptor usage, and secreted versus intracellular OPN. We then discuss its roles in CNS development, chronic neurological diseases, acute CNS injury, and neuro-oncology, and highlight the need to distinguish biomarker associations, omics-based candidate pathways, and functionally validated mechanisms when considering OPN-related diagnostic or therapeutic strategies.

## 1. Introduction

The SPP1/Spp1 gene encodes osteopontin (OPN), a secreted phosphoprotein originally identified as a bone matrix-associated protein and historically known as bone sialoprotein I and early T-lymphocyte activation-1 [1]. The term “osteopontin” reflects its initial association with bone matrix and adhesive extracellular matrix interactions. For clarity, SPP1 is used in this review to denote the human gene, Spp1 the mouse gene, and OPN the encoded protein. When transcriptomic or spatial-omics studies are discussed, we refer to SPP1/Spp1 expression or SPP1/Spp1-expressing cells, whereas OPN is used for protein localization, secretion, receptor engagement, and functional protein activity.

OPN has attracted increasing attention in neuroscience because it links extracellular matrix remodeling, immune regulation, and disease-associated cellular state transitions. In the central nervous system (CNS), OPN has been implicated in developmental tissue remodeling, white matter maturation, acute injury repair, chronic neuroinflammation, neurodegenerative disease, and brain tumor progression. Its function remains controversial. In developmental and acute injury settings, OPN has been associated with neuroprotection, tissue preservation, debris clearance, and regenerative responses. For example, Spp1-positive microglia participate in the repair of local developmental lesions and help protect the fetal brain from morphogenetic stress and injury [2]. CD11c-positive microglial populations can also produce OPN after apoptotic neuron clearance, linking developmental remodeling to microglial immunoreactivity [3]. In contrast, sustained or ectopic OPN activity has been associated with inflammatory persistence, synaptic injury, demyelination, and immunosuppressive tumor progression. CRISPRi/a-based screening identified Spp1 as a disease-associated microglial marker enriched across several neurological disease contexts, including Alzheimer’s disease, multiple sclerosis, aging, and glioma, and showed that Spp1 expression can be modulated by CSF1R or MAPK14 perturbation [4]. These findings support SPP1/Spp1 as a disease-associated marker and candidate regulatory node, but they do not by themselves establish OPN as a universally protective or harmful mediator.

The central premise of this review is that OPN-related effects in the CNS should be interpreted according to molecular form, cellular source, receptor engagement, anatomical compartment, disease stage, and strength of experimental evidence. During development or acute injury, transient OPN induction may support boundary maintenance, myelin-related maturation, debris clearance, barrier repair, angiogenesis, and tissue reconstruction [5,6]. In chronic inflammatory lesions or tumor-associated niches, prolonged OPN signaling may instead contribute to glial reactivity, remyelination failure, synaptic vulnerability, immune evasion, vascular adaptation, and invasive growth [7,8]. This distinction provides the conceptual basis for evaluating when OPN participates in reparative remodeling and when it may stabilize pathological persistence.

This review is primarily focused on OPN biology in the CNS and CNS-associated pathological compartments, including the brain, spinal cord, cerebrospinal fluid, and CNS tumor microenvironments. Peripheral OPN biology is discussed only when it directly influences CNS pathology, biomarker interpretation, or neuroimmune communication. Because OPN is also produced by peripheral immune cells, bone-related tissues, vascular cells, renal and urinary tract compartments, and tumor-associated tissues, circulating or urinary OPN should not be interpreted as CNS-derived without supporting evidence from CSF analysis, lesion localization, cellular-source mapping, or disease-context-specific validation. In addition, the depth of discussion varies across disease sections according to the amount and strength of available evidence. Greater emphasis is placed on contexts supported by mechanistic, clinical, biomarker, or multi-omics evidence, such as multiple sclerosis, Alzheimer’s disease, acute CNS injury, and glioblastoma, whereas conditions supported mainly by limited cohorts, transcriptomic association, spatial colocalization, or incompletely validated experimental models are discussed more briefly.

## 2. Molecular Basis of OPN Signaling

### 2.1. Molecular Structure, Isoforms, Post-Translational Processing, and Localization of OPN

OPN is a heterogeneous phosphoglycoprotein rather than a single uniform molecular species [9]. Its diversity arises from alternative splicing, alternative translation, post-translational modification, proteolytic processing, and differential subcellular localization. Human OPN contains 314 amino acids, although its apparent molecular weight varies substantially because of phosphorylation, glycosylation, sulfation, and protease-dependent cleavage. The SPP1 gene contains seven exons, with exons 2–7 encoding the protein-coding region. Alternative splicing generates several transcript variants, including OPN-a as the full-length isoform, OPN-b lacking exon 5, OPN-c lacking exon 4, OPN-4 lacking exons 4 and 5, and OPN-5 containing an additional exon derived from intron retention [10,11]. Although these splice variants have been studied mainly in cancer and peripheral tissues, they are relevant to CNS research because total OPN protein or SPP1/Spp1 expression does not necessarily represent a single functional molecular form.

Post-translational modification further increases OPN heterogeneity. Phosphorylation and glycosylation may influence protein conformation, extracellular matrix binding, receptor accessibility, and apparent molecular size. Proteolytic cleavage is particularly important because it can alter receptor usage [12]. Full-length OPN contains an RGD motif that mediates binding to several αv-containing integrins. Thrombin cleavage exposes the cryptic SVVYGLR sequence in the N-terminal fragment, thereby increasing accessibility to integrins such as α4β1, α9β1, and α4β7 [13]. Other proteases, including matrix metalloproteinases, plasmin, and cathepsins, can also generate OPN fragments with altered receptor-binding properties. Thus, increased total OPN in tissue, CSF, or plasma may reflect a mixture of full-length protein, cleaved fragments, and modified forms rather than a single signaling molecule.

The distinction between secreted and intracellular OPN adds another layer of complexity. Secreted OPN mainly functions as an extracellular matrix-associated ligand that engages integrins and CD44 on neighboring cells. Intracellular OPN should be considered a distinct functional form rather than a simple precursor of secreted OPN. In immune cells, secreted and intracellular OPN can arise from the same Spp1 transcript through alternative translation and may mediate different biological activities [14]. Intracellular OPN has been implicated in cytoskeletal organization and signaling downstream of innate immune receptors, whereas secreted OPN primarily acts through extracellular receptor engagement. Direct evidence for intracellular OPN in CNS diseases remains more limited than that for secreted OPN. Therefore, SPP1/Spp1 upregulation in transcriptomic datasets should not be assumed to represent a specific secreted or intracellular OPN function without protein localization, secretion, or functional data.

This molecular heterogeneity has direct implications for CNS disease interpretation and biomarker use. In many neurological studies, OPN is measured as total protein in tissue, CSF, plasma, or serum, while the specific splice variant or cleavage product is not identified. As a result, it is often difficult to assign a disease-associated effect to full-length OPN or to a defined proteolytic fragment unless form-specific antibodies, mass spectrometry, fragment-selective assays, or functional perturbation experiments are used. Accordingly, total OPN protein and SPP1/Spp1 expression should be interpreted as form-limited evidence unless the active molecular species is experimentally defined.

Secreted OPN has been detected in brain tissue, CSF, plasma, and serum, indicating that it may act both as a local extracellular ligand and as a compartment-dependent fluid biomarker. Elevated CSF OPN has been reported in neuroinflammatory diseases such as MS [15], AD-related cohorts [16], acute vascular injury such as subarachnoid hemorrhage [17], and CNS malignancies [18]. OPN changes have also been reported in neuroinfectious contexts, including HIV-associated CNS disease [19,20], cerebral malaria [21], cryptococcal meningitis [22], and human African trypanosomiasis [23], where brain, CSF, or circulating OPN may reflect infection-associated neuroinflammation, glial or myeloid activation, and compartment-specific host responses. These findings suggest that secreted OPN may reflect glial activation, macrophage or microglial remodeling, barrier disruption, vascular injury, or tumor-associated immune remodeling. However, CSF or plasma OPN should not be interpreted as a direct equivalent of lesion-derived OPN, because secreted OPN may originate from resident CNS cells, infiltrating immune cells, tumor cells, meningeal or perivascular compartments, and peripheral tissues [24]. Moreover, evidence based on SPP1/Spp1 transcript abundance, total OPN immunostaining, or CSF/serum OPN measurement usually cannot define the active OPN species. Brain and CSF OPN findings therefore require interpretation together with cellular source, lesion localization, receptor expression, disease stage, sample compartment, and, where possible, form-selective protein or functional validation.

### 2.2. The Receptor Axis of OPN

In CNS-related studies, OPN signaling has mainly been interpreted through two receptor systems: integrins and CD44. Integrin-dependent signaling, particularly through αv-containing complexes, links OPN to focal adhesion signaling, cytoskeletal remodeling, migration, adhesion, phagocytosis, survival, and angiogenic responses [25,26]. These effects are commonly associated with downstream pathways such as FAK/Src, ERK, and PI3K/AKT. Because OPN cleavage can expose cryptic binding motifs, proteolytic processing may broaden integrin engagement and alter downstream signaling outcomes. This provides one molecular explanation for why full-length and cleaved OPN may not produce equivalent biological effects.

CD44-dependent OPN signaling is more closely linked to cell-state transitions and inflammatory or tumor-associated remodeling [27]. In demyelinating disease, epilepsy, stroke, and brain tumors, OPN-CD44 signaling has been implicated in astrocyte activation, OPC responses, inflammatory persistence, tumor-cell plasticity, and macrophage-associated remodeling [28,29,30,31]. However, CD44 exists as a standard isoform and multiple variant isoforms generated by alternative splicing, and these isoforms may differ in ligand binding, co-receptor interactions, and disease relevance. Most CNS studies do not define which CD44 isoform is involved or whether full-length OPN, cleaved OPN, or another modified form is the dominant ligand. Therefore, OPN-CD44 signaling should be interpreted according to the available evidence, especially when conclusions are based on transcriptomic or ligand–receptor inference rather than direct receptor-level perturbation.

Overall, receptor usage provides a key link between OPN molecular heterogeneity and disease-specific function. Integrin-related pathways are more often associated with adhesion, migration, phagocytosis, survival, and vascular remodeling, whereas CD44-related pathways are more frequently connected to glial state transitions, inflammatory persistence, and tumor-associated plasticity [32]. This receptor framework provides the basis for the disease-specific interpretations discussed below.

### 2.3. Determinants of Reparative Versus Pathological OPN Signaling

The central thesis of this review is that OPN activity in the CNS should not be interpreted as uniformly protective or detrimental. Rather, its biological outcome depends on the interaction among molecular form, receptor engagement, producing cell type, target cell state, anatomical compartment, and disease stage. This framework is particularly important because increased SPP1/Spp1 expression or total OPN protein does not necessarily indicate a single functional role [27].

In developmental and acute injury settings, OPN induction is often coupled to controlled tissue remodeling, including microglial clearance, vascular stabilization, barrier repair, and glial or axonal repair [2,33,34]. In contrast, when OPN activity persists in chronically inflamed lesions or tumor-associated niches, it may contribute to maladaptive remodeling by sustaining glial reactivity, impairing remyelination, promoting synaptic vulnerability, or reinforcing an immunosuppressive microenvironment [28,31,35]. Thus, the shift from reparative to pathological OPN activity is best understood as a stage- and compartment-dependent process rather than as a simple consequence of increased expression.

This functional divergence is also shaped by OPN form and receptor usage [36]. Secreted full-length OPN mainly acts as an extracellular matrix-associated ligand, whereas proteolytic cleavage can expose additional integrin-binding motifs and alter receptor preference. Intracellular OPN may represent a distinct signaling mode, particularly in immune cells. Integrin-linked OPN signaling is commonly associated with adhesion, migration, phagocytosis, survival, and vascular remodeling, whereas CD44-related signaling has been implicated in glial state transitions, inflammatory persistence, remyelination failure, and tumor-associated plasticity. Together, these determinants provide the conceptual basis for interpreting OPN-related findings in development, chronic neurological disease, acute CNS injury, and neuro-oncology.

## 3. Spp1/OPN in CNS Development

**Figure 1:**
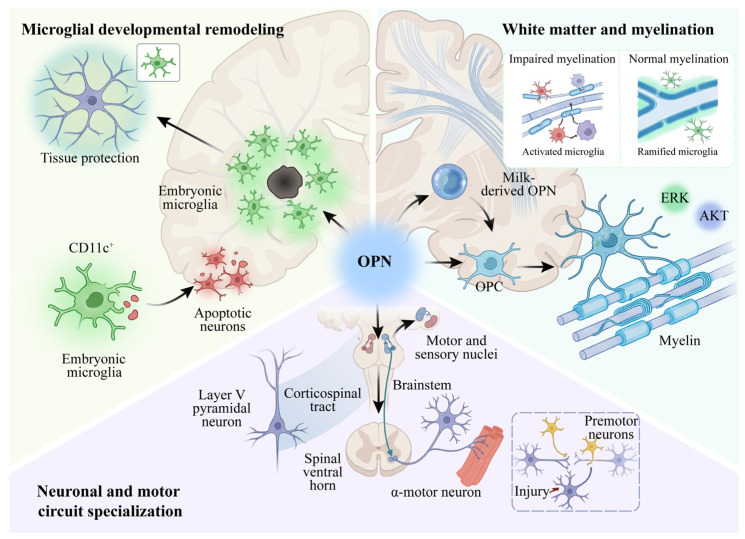
Spp1/OPN-related processes in CNS development.

The developmental stage provides a crucial foundation for studying the role of OPN in the CNS. During development, OPN is primarily involved in physiological tissue remodeling, microglial function, white matter maturation, myelination, and the establishment of specific neural circuits. These findings suggest that Spp1/OPN may contribute to developmental tissue maintenance, microglial remodeling, and circuit-associated maturation in a context-dependent manner (Figure 1).

During CNS development, Spp1/OPN is associated with microglial developmental remodeling, white matter maturation, myelination, and neuronal or motor circuit specialization. In the embryonic brain, Spp1-positive microglia accumulate at structurally vulnerable regions and contribute to tissue protection and local lesion repair. CD11c^+^ microglial subpopulations can produce OPN following apoptotic neuron clearance, suggesting a link between developmental phagocytosis and microglial remodeling states. Milk-derived OPN may enter the developing brain and promote oligodendrocyte precursor cell (OPC) differentiation and myelin-related maturation through ERK and PI3K–AKT signaling. Spp1 expression has also been reported in selected motor and sensory nuclei, layer V corticospinal neurons, and spinal α-motor neurons, and may increase in premotor neurons after corticospinal tract injury. Arrows summarize OPN- or Spp1-related relationships described in the cited studies.

### 3.1. The Role of OPN in Maintaining the Function and Structure of Developing Microglia

Spp1/OPN is involved in tissue protection and repair programs mediated by developing microglia. At the junctions of structures growing in different directions within the fetal cortex, embryonic microglia aggregate and exhibit a phenotype similar to that of postnatal axon-associated microglia, with Spp1 expression being one of the associated features. Spp1 helps prevent the progression of microcavities into larger cavitary lesions and promotes rapid repair of local lesions [2]. Therefore, OPN may assist developmental microglia in maintaining the structural integrity of the fetal brain and responding to physiological stress and microinjuries during morphogenesis. Perinatal studies have further expanded this understanding. A stable subpopulation of CD11c-positive microglia has been identified as capable of producing OPN; these cells emerge following the phagocytosis of apoptotic neurons and persist into adulthood [3]. These findings suggest that OPN is not merely a marker of transient activation but may characterize a developmental microglial state with tissue-remodeling and repair-associated features. Furthermore, brain injury models in early life suggest that the Spp1-associated microglial state exhibits significant age-dependence. In neonatal brain injury, Spp1^+^ microglia do not represent a terminal, fixed pathological state; rather, they can regain homeostatic characteristics and reintegrate into the microglial population during the recovery process. In contrast, following injury during childhood, similar Spp1^+^ microglia are more likely to enter an irreversible trajectory and are ultimately cleared [6]. Thus, the developmental timing not only determines whether OPN is induced but also influences the subsequent stability and functional trajectory of the cellular state it marks.

### 3.2. The Role of OPN in White Matter Development and Myelination

OPN is associated with white matter maturation and developmental myelination. Lactogenic OPN can enter brain tissue early in life and increase intracellular OPN levels, thereby promoting oligodendrocyte lineage development, myelin-associated protein expression, and ERK/PI3K-AKT pathway activation [5]. These findings suggest that OPN may support early brain maturation and myelin-related developmental processes. However, spatial transcriptomic studies of human developing white matter have shown that Spp1 is also upregulated in regions with impaired developmental myelination and is associated with focal immune dysregulation involving microglia/macrophages and type II interferon signaling [37]. In this context, Spp1 may serve as a marker linking abnormal microglial or macrophage-associated immune states to impaired myelination. Thus, OPN in developing white matter should not be interpreted only as a signal of successful maturation; it may also reflect stress-associated glial and immune remodeling. Overall, OPN-related signaling during white matter development may participate in oligodendrocyte lineage progression, local immune regulation, and glial state transitions.

### 3.3. The Role of OPN in Neurons and Motor Circuits

Spp1 expression is not restricted to glial-associated developmental remodeling but is also detected in selected neuronal populations within motor-related circuits. OPN has been reported in specific motor and sensory nuclei of the rat hindbrain and in cerebellar-associated neurons [38]. In primates, Spp1 is highly expressed in large layer V pyramidal neurons of the sensorimotor cortex, with a distribution corresponding closely to corticospinal neurons [39]. Spp1 is also selectively expressed in motor neurons of lamina IX in the anterior horn of the macaque spinal cord, with expression patterns related to neuronal size and segmental distribution, whereas weaker expression has been observed in presumed γ-motor neurons and in the Onuf nucleus [40]. Spp1-positive neurons are more abundant in primate species with more developed corticospinal systems and increase gradually during postnatal development. After corticospinal tract injury, Spp1-positive neurons in the premotor cortex are upregulated during functional recovery [41]. These findings suggest that Spp1 may be associated with neuronal subtype identity, maturation, and circuit specialization in corticospinal neurons and large α-motor neurons, particularly within highly developed primate motor systems.

## 4. The Role of Spp1/OPN in Chronic CNS Diseases

**Figure 2:**
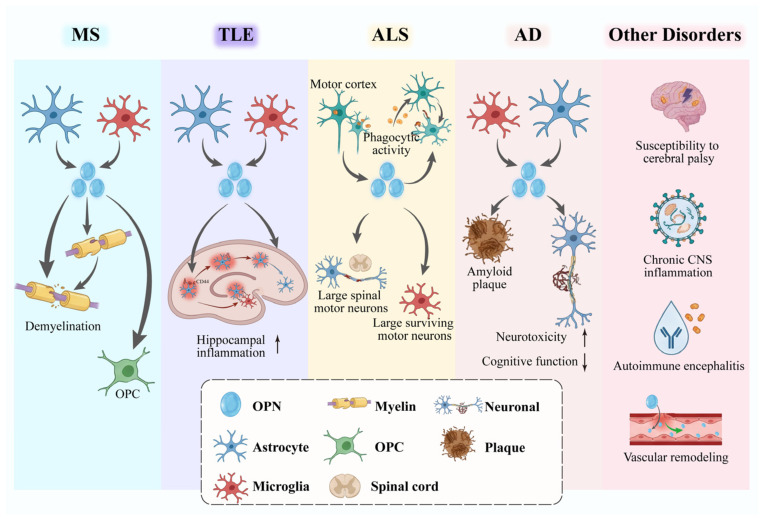
Spp1/OPN-related processes in chronic CNS diseases.

In chronic CNS diseases, SPP1/Spp1 expression and OPN protein changes are frequently associated with persistent neuroinflammation, glial state transitions, tissue stress, and repair-related remodeling. However, the interpretation of OPN differs across disease contexts and depends on whether the evidence reflects transcriptomic association, protein-level biomarker change, or functional perturbation (Figure 2).

In multiple sclerosis, Spp1/OPN is associated with astrocyte-related white matter pathology and compartmentalized inflammation, and astrocyte-derived Spp1/OPN can inhibit oligodendrocyte precursor cell (OPC) progression and remyelination through CD44-related signaling. In temporal lobe epilepsy, Spp1 is induced in reactive glial populations and has been proposed as part of a candidate microglia–astrocyte communication axis associated with hippocampal inflammation. In amyotrophic lateral sclerosis, OPN is enriched in selected motor neuron subpopulations and, as disease progresses, is associated with astrocyte migration and microglial phagocytic activity. In Alzheimer’s disease, Spp1/OPN is linked to plaque-associated myeloid states, perivascular immune remodeling, amyloid-related responses, and context-dependent effects on synaptic remodeling or amyloid clearance. Spp1/OPN has also been implicated in other chronic CNS-related disorders, including cerebral palsy, CNS neuroinflammation, autoimmune encephalitis, and vascular remodeling-related conditions. Arrows summarize OPN- or Spp1-related relationships described in the cited studies. Upward and downward arrows indicate increased and decreased changes in the indicated cellular, pathological, or functional readouts, respectively.

### 4.1. OPN in Multiple Sclerosis and Demyelinating Diseases

In multiple sclerosis (MS), Spp1 expression is not confined to active demyelinating lesions. It is also upregulated in normal-appearing white matter and is primarily localized to astrocytes, suggesting that it may reflect widespread glial stress and tissue remodeling in MS [42]. CSF studies have further shown that OPN protein levels are associated with persistent intrathecal inflammatory activity. In primary progressive MS, elevated CSF OPN has been linked to early intracerebral innate immune and glial activation [43]. In early-stage MS, higher CSF OPN levels are associated with cortical atrophy, increased disease activity, and disability progression [44], supporting its potential value for disease stratification and prognostic assessment. A recent systematic review and meta-analysis also supports the biomarker relevance of OPN in MS, reporting associations between CSF or peripheral blood OPN levels and MS diagnosis, clinical phenotype, and treatment-response assessment, although substantial heterogeneity among cohorts and assay platforms remains [45]. At the lesion level, Spp1 is associated with the transcriptional profile of brain-resident memory T cells and the compartmentalized immune environment within MS lesions. T cells isolated from MS lesions exhibit reduced inflammatory cytokine production after stimulation, suggesting that Spp1-associated signals may contribute to the local adaptive immune milieu within the CNS [46]. These observations support OPN as a marker of glial stress, compartmentalized inflammation, and lesion-associated immune remodeling, but they do not by themselves establish OPN as a direct driver of demyelination or remyelination failure.

By contrast, astrocyte-derived OPN acting through CD44 expressed on oligodendrocyte precursor cells (OPCs) represents a stronger mechanistic example. In this setting, Spp1/OPN-CD44 signaling inhibits oligodendrocyte lineage progression and remyelination, whereas targeting astrocyte CLC2/CLCN2 restores myelin regeneration by reducing this pathway [28]. Thus, in MS and demyelinating disease, OPN should be interpreted in two related but distinct ways: as a marker of glial stress or inflammatory lesion activity in broader observational studies, and as a potential driver of impaired remyelination when supported by cell-source-specific and receptor-linked functional evidence.

Most MS studies have measured total OPN in tissue or CSF rather than distinguishing full-length OPN from thrombin- or MMP-cleaved fragments. Therefore, the contribution of specific OPN fragments to CD44- or integrin-mediated remyelination failure remains unresolved.

### 4.2. The Role of OPN in Temporal Lobe Epilepsy and Seizures

Spp1 expression has been identified as a representative feature of epilepsy-associated glial activation in temporal lobe epilepsy (TLE). scRNA-seq, snRNA-seq, and Xenium spatial transcriptomic analyses have shown that Spp1 is upregulated in multiple glial populations, including microglia, astrocytes, oligodendrocytes, and OPCs, suggesting that SPP1/Spp1-expressing glial states may mark hippocampal inflammation and altered cellular interaction networks [47]. Multicohort snRNA-seq analysis of human surgical specimens further suggested that Spp1 may mark or participate in inflammatory communication between reactive microglia and astrocytes in TLE. In particular, integrated single-nucleus and spatial transcriptomic analyses support a candidate Spp1-CD44 communication axis between reactive microglia and astrocytes. However, because this conclusion is mainly based on expression patterns and inferred ligand–receptor interactions, direct functional validation using Spp1 perturbation, OPN protein-level assessment, or CD44 blockade is still needed to determine whether OPN actively drives the inflammatory cascade [29]. Thus, in TLE, Spp1 should currently be interpreted mainly as an inflammation-associated glial marker and candidate component of abnormal microglia–astrocyte communication, rather than as a fully validated causal driver.

### 4.3. The Role of OPN in Amyotrophic Lateral Sclerosis

In amyotrophic lateral sclerosis (ALS), OPN has been reported in relatively resilient motor neuron subpopulations and may be associated with motor neuron subtype vulnerability. As the disease progresses, extracellular OPN accumulation has been linked to astrocyte migration and microglial phagocytic responses through αvβ3 integrin/MMP-9 and CD44-related pathways [48]. These findings suggest that OPN may participate in both compensatory cellular remodeling and late-stage pathological remodeling, although its precise causal role in ALS progression remains incompletely defined. Spp1 is normally expressed in large pyramidal neurons of the sensorimotor cortex and in motor neurons of the spinal cord anterior horn, but its expression is reduced in ALS patients, particularly in the largest surviving neurons [49]. Overall, Spp1 expression appears to mark selected motor neuron subpopulations and may also be associated with glial and extracellular remodeling during ALS progression.

### 4.4. The Role of OPN in Alzheimer’s Disease and Related Chronic Degenerative Diseases

In Alzheimer’s disease (AD), SPP1/Spp1 expression and OPN protein changes have been linked to plaque-associated myeloid states, perivascular immune remodeling, and amyloid-related responses. Existing studies indicate that Spp1 derived from perivascular macrophages and fibroblasts can induce hippocampal microglia to upregulate phagocytic markers such as C1qa, Grn, and Ctsb. In AD mouse models, the absence of Spp1 prevents synaptic loss [7,50]. Spp1 is also one of the representative molecules of reactive microglial subpopulations associated with Aβ plaques, and its expression is regulated by ApoE, suggesting that Spp1 marks an AD-associated microglial state linked to both tissue repair and potential pathological remodeling [51]. Multi-omics studies have further shown that Spp1-associated signals are detected in CSF, cortical tissue, and peripheral fluids and are associated with neuropathology and immune activation [52,53].

These findings should be interpreted according to evidence level and disease context. Increased Spp1 expression in plaque-associated microglia or perivascular macrophage-like populations may indicate a disease-associated phagocytic and inflammatory state, but expression alone does not prove that OPN directly drives amyloid clearance, synaptic engulfment, or neurotoxicity. In MAPT-N279K-associated frontotemporal dementia and obesity-related brain inflammation, Spp1 has been associated with neuronal abnormalities, peripheral myeloid cell infiltration, and pro-inflammatory remodeling [54,55]. By contrast, during lecanemab treatment or peripheral macrophage-mediated Aβ clearance, OPN-related responses may support amyloid clearance and tissue remodeling [56,57]. Human iPSC-based tri-culture models and single-cell integrative analyses further suggest that Spp1 is co-regulated by astrocytes, diseased neurons, and the local metabolic environment [58,59].

Therefore, Spp1/OPN in AD should be interpreted in two related but distinct ways. It may serve as a marker of plaque-associated microglial or perivascular myeloid activation, while functional involvement in synaptic remodeling, amyloid clearance, or neurotoxicity requires direct experimental support. Beneficial effects are more likely to be observed in acute or treatment-associated amyloid clearance contexts, whereas detrimental effects may arise when Spp1-positive microglial, perivascular, or stromal-associated states are chronically sustained. Whether these effects are mediated by full-length OPN, cleaved OPN fragments, intracellular OPN, or specific receptor pathways remains unresolved in most AD studies.

### 4.5. Brief Evidence from Other CNS-Related Chronic Conditions

OPN has also been reported in several other chronic CNS-related disorders, although the available evidence is generally more limited and disease-specific. Genetic studies suggest that SPP1/Spp1-related polymorphisms may be associated with susceptibility to cerebral palsy in the Han Chinese population; specifically, rs1126616 was significantly associated with overall cerebral palsy, suggesting that OPN-related pathways may be linked to inflammatory or neuroprotective responses after perinatal brain injury [60]. In autoimmune encephalitis, elevated CSF and serum OPN levels correlate with disease severity and imaging abnormalities, supporting its potential value as a biomarker of active CNS immunopathology [61]. In cerebral amyloid angiopathy, cerebral small vessel disease, and related vascular cognitive disorders, SPP1/Spp1 expression or OPN-associated signals have been linked to vascular wall remodeling, calcification, and white matter lesions [62,63]. Together, these observations suggest that OPN may be associated with glial activation, neuronal injury, vascular remodeling, and local immune responses in selected chronic CNS conditions. However, in many of these contexts, the evidence remains mainly genetic, biomarker-based, or correlative, and disease-specific functional validation is still needed.

### 4.6. OPN in Neuroinfectious Diseases and Infection-Associated Neuroinflammation

Neuroinfectious diseases provide another important context in which SPP1/Spp1 expression and OPN protein changes should be interpreted according to cellular source, anatomical compartment, and disease stage. Infection-associated neuroinflammation can overlap with several pathological processes discussed above, including glial activation, myeloid recruitment, vascular dysfunction, synaptic injury, demyelination-related stress, and long-term cognitive impairment. Therefore, OPN in neuroinfectious disease may represent not only an inflammatory biomarker but also a potential regulator of host–pathogen interaction and CNS immune remodeling [21,64].

In HIV-associated CNS disease, OPN has been detected in brain tissue and CSF and has been linked to macrophage or microglial activation, viral replication, and neurocognitive impairment [19,20]. OPN is increased in the brains of patients with HIV encephalitis and in SIV encephalitis models, and CSF OPN levels are elevated in HIV-infected individuals [19]. Additional work showed that OPN can enhance HIV replication in macrophage-related systems and is increased in the brain and CSF of HIV-infected individuals [20]. However, OPN may not act uniformly as a pro-inflammatory mediator in chronic viral infection. Experimental work from chronic CNS viral infection models suggests that OPN/SPP1 can also behave as a molecular brake on neuroinflammatory responses [64].

In addition, work from the Brown laboratory has suggested that cortical neurons can be a prominent OPN source in HIV/SIV-associated CNS disease, indicating that infection-related OPN should not be attributed exclusively to myeloid cells [65]. These findings support a context-dependent interpretation in which OPN may mark HIV/SIV-associated myeloid activation and CNS inflammation, while its functional consequences depend on viral persistence, macrophage or microglial state, and local immune regulation.

Evidence from parasitic and fungal CNS infections further supports the relevance of OPN in neuroinfectious inflammation. In cerebral malaria, OPN levels are elevated in both plasma and CSF compared with non-cerebral malaria neurological infections, suggesting that OPN may reflect a compartmentalized inflammatory response associated with neurological involvement [21]. In human African trypanosomiasis, OPN and β2-microglobulin have been identified as candidate biomarkers for disease staging, supporting the potential value of OPN-related measurements in infection-associated CNS involvement [23]. In cryptococcal meningitis, recent experimental evidence indicates that delayed microglial activation is accompanied by microglial osteopontin/Spp1 responses and that microglial Spp1 may impair peripheral host control [22]. This suggests that infection-induced OPN responses may sometimes worsen host–pathogen balance rather than simply promote repair.

OPN has also been investigated in systemic infectious or post-infectious inflammatory contexts relevant to neurological interpretation, including COVID-19 and sepsis. Circulating OPN levels have been associated with disease severity, mechanical ventilation, or mortality in hospitalized COVID-19 patients [66] and have also been studied as a systemic inflammatory and prognostic marker in SIRS, sepsis [67], and septic shock [68]. These findings should be interpreted cautiously in a CNS-focused review because plasma or serum OPN is not CNS-specific and may be influenced by peripheral immune activation, vascular injury, renal function, tissue remodeling, sample matrix, and assay platform [69,70]. Overall, neuroinfectious diseases reinforce the central framework of this review: OPN may function as a compartment-sensitive inflammatory and remodeling signal, but its biological meaning depends on whether it is measured in brain tissue, CSF, plasma, or serum, and whether the evidence reflects biomarker association, cellular-source mapping, or direct functional perturbation.

## 5. Spp1/OPN in Acute Central Nervous System Injury

**Figure 3:**
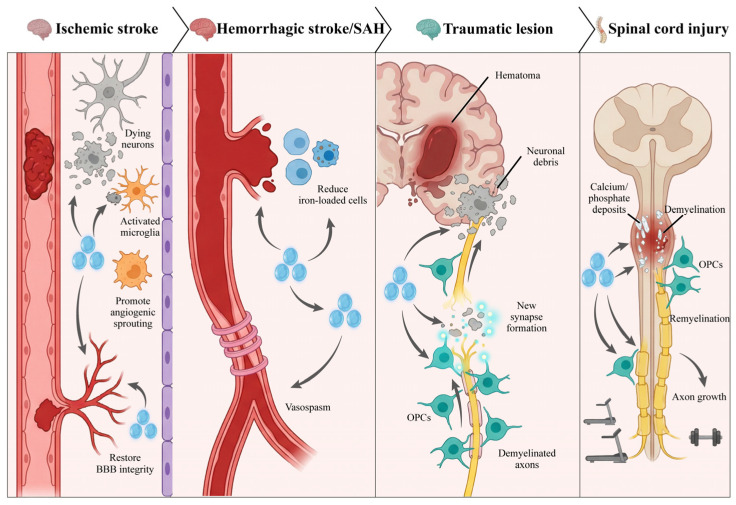
Spp1/OPN-related processes in acute CNS injury.

Compared with chronic neurological diseases, acute CNS injury provides a useful setting for understanding the temporal and spatial transition of OPN activity. After ischemic stroke, hemorrhagic stroke, traumatic brain injury (TBI), or spinal cord injury, OPN is commonly induced in activated microglia, macrophages, reactive glia, vascular-associated cells, and lesion-associated extracellular matrix compartments [71,72]. During the early injury phase, this response may contribute to debris clearance, blood–brain barrier or blood-spinal cord barrier repair, vascular remodeling, angiogenesis, and tissue reconstruction [33,34]. However, as injury evolves, sustained OPN expression at chronically inflamed lesion borders, white matter injury zones, or glial scar-associated compartments may be associated with inflammatory persistence, ferroptosis-related injury, demyelination, scar-associated remodeling, and impaired tissue recovery (Figure 3). Therefore, in acute CNS injury, OPN should be interpreted according to both disease stage and lesion compartment, rather than as a uniformly reparative or detrimental molecule.

Proteolytic processing may be particularly relevant in acute CNS injury, because thrombin, matrix metalloproteinases, plasmin, and other proteases are dynamically activated after ischemic, hemorrhagic, traumatic, or spinal cord injury [13,73,74]. However, most available studies still measure total OPN or SPP1/Spp1 expression rather than defined OPN fragments, limiting direct assignment of reparative or pathological effects to a specific OPN species.

In acute CNS injury, Spp1/OPN shows stage- and compartment-dependent associations with tissue remodeling. In ischemic stroke, OPN is induced in activated microglia and myeloid cells within the infarct core or perilesional regions and is associated with debris clearance, blood–brain barrier repair, angiogenic remodeling, and recruitment of reactive glial cells or oligodendrocyte precursor cells (OPCs), but may also contribute to inflammatory amplification and secondary tissue injury in specific lesion contexts. In hemorrhagic stroke and subarachnoid hemorrhage, OPN is linked to vascular protection, blood–brain barrier stabilization, vasospasm attenuation, and reduction of secondary injury. In traumatic brain injury, OPN is associated with microglial activation, debris clearance, synaptic remodeling, and glial scar-related responses. In spinal cord injury, OPN is involved in post-injury vascular remodeling and may enhance rehabilitation-related plasticity, thereby supporting functional recovery and partial axonal regeneration. Arrows summarize OPN- or Spp1-related relationships described in the cited studies.

### 5.1. OPN in Ischemic Stroke

At 3–7 days after cerebral ischemia–reperfusion, Spp1 expression is enriched mainly in activated microglia within the infarct core [75] and strongly colocalizes with calcium and phosphorus deposits surrounding neuronal debris [76], suggesting a possible role in the recognition and clearance of necrotic or apoptotic cellular debris. Single-cell and spatial-omics studies further indicate that Spp1 is expressed by perilesional myeloid cells and identify CD44 as a candidate receptor through which Spp1/OPN-related signaling may influence the recruitment of reactive astrocytes and OPCs to the injury margin [77]. Furthermore, the Spp1-associated microglial state exhibits distinct age- and fate-dependent characteristics. Spp1^+^ DAM-like cells formed after stroke in newborns can revert to a steady state after recovery, whereas a similar state induced by injury during childhood is more likely to enter an irreversible trajectory and eventually disappear [6].

However, some studies suggest that Spp1/OPN may also exacerbate ischemic brain injury. Spp1/OPN inhibition reduces infarct volume, oxidative stress, and neurological dysfunction [78]. The Spp1-Cd44 axis has been proposed as a candidate communication pathway between activated microglia, peripheral immune cells, and local glial cells, based mainly on spatial transcriptomic and ligand–receptor inference. Its direct functional contribution to post-stroke inflammatory amplification requires further validation through Spp1/OPN or CD44 perturbation [30]. In pontine infarction, a subset of Spp1^+^ microglia interacts with oligodendrocytes and is associated with demyelination; inhibiting the proliferation of these cells improves tissue damage and neurological function [79]. Therefore, OPN-related effects in ischemic stroke may differ between the infarct core, where debris recognition and clearance dominate, and the perilesional zone, where reactive astrocytes, OPCs, infiltrating immune cells, and vascular remodeling determine whether OPN-associated signaling supports repair or amplifies secondary injury.

### 5.2. OPN in Hemorrhagic Stroke and Subarachnoid Hemorrhage

In hemorrhagic injury, OPN has been implicated in inflammatory regulation, vascular remodeling, and barrier repair. Macrophage-derived OPN promotes astrocyte polarization and supports blood–brain barrier (BBB) reconstruction following vascular injury in stroke [80]. In subarachnoid hemorrhage (SAH), OPN has been associated with vasoprotective effects in several experimental contexts. OPN upregulation can mitigate BBB disruption and alleviate cerebral vasospasm through CD44/P-gp signaling [81,82], suggesting a role in vascular stabilization and early brain injury attenuation after SAH. In intracerebral hemorrhage, Spp1/OPN has been reported to reduce ferroptosis and oxidative damage through Nrf2/HO-1 and BDNF-related signaling [83,84], thereby alleviating hippocampal inflammation, structural injury, depression-like behavior, and cognitive impairment after hemorrhagic injury. Together, these findings suggest that OPN in hemorrhagic CNS injury is closely linked to vascular and barrier-associated compartments. Its function may vary according to whether it is induced during early vascular stabilization, iron-related secondary injury, or later inflammatory remodeling.

### 5.3. OPN in Traumatic Brain Injury

In the early stages of traumatic brain injury (TBI), Spp1 expression is associated with microglial activation and inflammatory amplification. Its levels decrease significantly after inhibition of inflammation-related pathways such as CSF1R, IL1R1, or 5-LOX, accompanied by reduced tissue damage and improved neurological function [85]. In the olfactory bulb after mild TBI, OPN may also be linked to MMP9-CD44-related synaptic remodeling [86,87]. Focal toxic injury models further suggest that OPN is associated not only with debris clearance but also with astrocyte border formation and scar-related remodeling [72,88]. Thus, OPN in TBI appears to span both injury-resolution and scar-associated phases: early expression is linked to microglial/macrophage activation and debris clearance, whereas later or sustained expression may be associated with astrocyte border formation, extracellular matrix remodeling, and persistent glial reactivity.

### 5.4. OPN in Spinal Cord Injury

Following spinal cord injury, OPN should be interpreted in relation to the spatial organization of the lesion. In the lesion core, OPN may be associated with infiltrating macrophages, activated microglia, vascular remodeling, and extracellular matrix reorganization [89]. These responses may contribute to damaged tissue clearance and formation of a provisional repair matrix, but sustained macrophage or microglial activation may also support inflammatory persistence. In the perilesional region, OPN may influence astrocyte reactivity, glial scar formation, angiogenesis, and axonal remodeling [90]. The glial scar is not simply inhibitory, because it can restrict lesion expansion while also limiting axonal regrowth depending on its cellular and matrix composition. Therefore, OPN-associated signaling in this region may have dual implications for tissue containment and regenerative failure.

OPN may also participate in vascular and rehabilitation-related plasticity after spinal cord injury. Single-cell sequencing has revealed injury-associated endothelial cell subpopulations linked to angiogenesis, and microglia or macrophages may interact with these endothelial cells through Spp1-related signaling to support endogenous vascular remodeling [91]. In incomplete cervical spinal cord injury, treadmill training increases BDNF and IGF-1 expression and activates mTOR signaling, while OPN further amplifies this response and promotes p-S6 upregulation, functional recovery, and partial axonal regeneration [92]. These findings suggest that OPN effects may differ between the lesion core, perilesional scar border, and remote spinal segments engaged by activity-dependent plasticity.

Recent evidence from a thoracic spinal cord contusion model further supports the relevance of remote spinal segments, showing increased OPN expression in large neurons of the lumbar spinal cord and increased parvalbumin/OPN co-expression in interneurons within lamina IX of the ventral horn after injury [93]. This suggests that injury-induced OPN responses may extend beyond the lesion epicenter and may involve neuronal and interneuronal remodeling in locomotor-related spinal circuits. Together, current evidence supports a compartment-dependent view of OPN in spinal cord injury, involving macrophage/microglial remodeling, endothelial signaling, astrocyte scar regulation, extracellular matrix remodeling, and axon-supportive plasticity.

## 6. The Role of OPN in Neuro-Oncology

**Figure 4:**
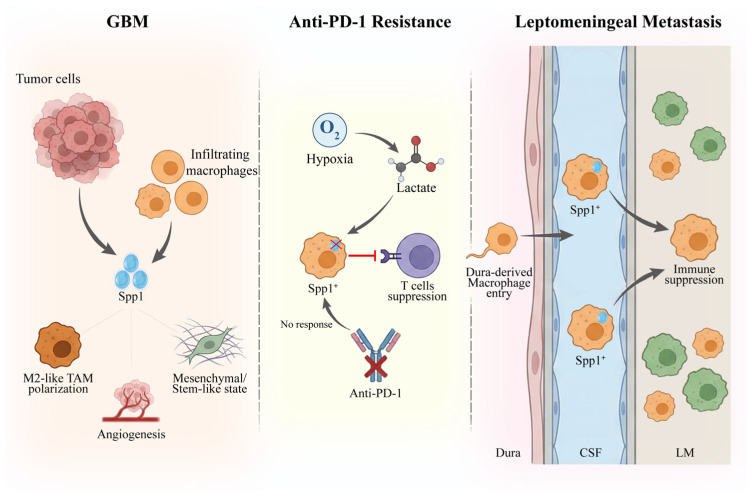
Spp1/OPN-related processes in neuro-oncology and immunotherapy resistance.

In glioblastoma (GBM) and brain metastases, OPN is integrated into reciprocal tumor–myeloid interactions associated with immune evasion, vascular adaptation, and invasive growth (Figure 4).

In glioblastoma (GBM), Spp1/OPN is linked to reciprocal interactions between tumor cells and myeloid cells. Tumor-cell-derived or macrophage-associated OPN is associated with macrophage recruitment, immunosuppressive tumor-associated macrophage (TAM) states, angiogenic remodeling, and stem-like or mesenchymal-like tumor phenotypes. In hypoxic tumor contexts, lactate-associated remodeling has been linked to the accumulation of SPP1/Spp1-positive macrophage states, which are associated with T-cell suppression and reduced responsiveness to anti-PD-1 therapy. In leptomeningeal metastasis, border- or dura-associated macrophages can enter the cerebrospinal fluid (CSF) compartment and contribute to an immunosuppressive CSF microenvironment that supports metastatic progression. Arrows summarize OPN- or Spp1-related relationships described in the cited studies.

### 6.1. OPN in GBM

In GBM, SPP1/Spp1 expression and OPN signaling have been closely linked to reciprocal interactions between tumor cells and myeloid cells. Existing studies indicate that OPN derived from tumor cells or infiltrating macrophages may promote macrophage recruitment and support the formation of immunosuppressive tumor-associated macrophage (TAM) states, thereby contributing to tumor-cell survival, angiogenesis, and malignant progression [35]. This process has been associated with macrophage-mediated tumor support in the context of PTEN loss [94], and with M2-like macrophage polarization induced by CEBPB-positive tumor-cell subpopulations [95]. In grade 2 and 3 gliomas, increased SPP1-positive TAMs are associated with T-cell exhaustion, tumor recurrence, and poorer survival [96]. These findings support SPP1-positive TAMs as an important myeloid state associated with glioma progression, although the strength of evidence differs across studies.

OPN derived from tumor cells may also contribute to tumor-cell plasticity and niche remodeling. In GBM, OPN has been associated with stemness-related phenotypes, perivascular niche remodeling, extracellular matrix interactions, and regulation of surrounding glial and macrophage states [97,98,99]. Thus, OPN-related signaling in GBM should be interpreted as part of a reciprocal tumor–myeloid interaction rather than as a single linear pathway. Tumor-cell-derived OPN may support macrophage recruitment and immunosuppressive TAM remodeling, whereas macrophage-derived OPN may further reinforce tumor-cell survival, angiogenic remodeling, and mesenchymal-like or stem-like programs. These effects are closely linked to receptor context. OPN-CD44 signaling has been associated with glioma cell plasticity, hypoxic adaptation, and stemness-related phenotypes, whereas OPN-integrin signaling, including ITGA5-related pathways, may contribute to macrophage remodeling, T-cell dysfunction, and immune escape [100]. Therefore, OPN may connect several malignant features of GBM, including myeloid immunosuppression, vascular adaptation, tumor-cell plasticity, and immunotherapy resistance.

In glioma studies, OPN signaling is often inferred from SPP1/Spp1 expression, total OPN protein, or ligand–receptor analysis. Whether distinct OPN splice variants or cleavage products differentially regulate CD44-, integrin-, or ITGA5-related tumor–myeloid communication remains unresolved.

### 6.2. OPN in Drug Resistance

The OPN-associated tumor microenvironment is also closely linked to treatment response. In brain tumors adapting to hypoxia, lactate-associated epigenetic remodeling can induce SPP1/Spp1-positive macrophage states, and macrophage-specific OPN deficiency improves the response to anti-PD-1 therapy [8]. Consistent with this finding, studies of immunotherapy resistance indicate that SPP1-positive myeloid macrophages are enriched in GBM samples that fail to respond to anti-PD-1 therapy. Single-cell and spatial transcriptomic analyses showed that these macrophages are located close to GBM cells and are associated with T-cell functional suppression through OPN-ITGA5-related signaling. After ITGA5 inhibition, the number of SPP1/Spp1-positive macrophages decreases and the efficacy of anti-PD-1 therapy improves, providing stronger functional support for the OPN-ITGA5 axis in therapy resistance than ligand–receptor inference alone [100]. Thus, OPN-related myeloid signaling may link immunosuppressive macrophage remodeling to treatment resistance in GBM.

The therapeutic implications of this pathway should nevertheless be interpreted cautiously. Direct systemic inhibition of OPN may be less specific than targeting disease-relevant signaling modules, because OPN can be produced by tumor cells, infiltrating macrophages, and other stromal or vascular-associated cells and also participates in tissue repair, vascular remodeling, and immune homeostasis in non-tumor settings. In GBM and metastatic CNS niches, more realistic strategies may include selective modulation of SPP1/Spp1-positive macrophage states, blockade of OPN-CD44 signaling involved in tumor-cell plasticity, or inhibition of OPN-ITGA5 signaling associated with T-cell suppression and anti-PD-1 resistance. These approaches require careful validation of disease stage, cellular source, receptor expression, and potential effects on physiological repair responses. Therefore, therapeutic development should avoid broad OPN blockade and instead focus on stage-specific, cell-state-specific, and receptor-axis-specific intervention strategies.

### 6.3. OPN in Brain Metastases and Meningeal Metastases

Single-cell studies of advanced non-small cell lung cancer (NSCLC) indicate that different metastatic sites are associated with distinct stromal and immune ecosystems. Although bone metastases have been linked to antigen-presenting cancer-associated fibroblast subsets and SPP1-related stromal interactions, CNS metastases show a different pattern in which SPP1 is more closely associated with macrophage-rich and immunosuppressive niches [101]. In leptomeningeal metastasis (LM), SPP1/OPN may contribute to the formation of an immunosuppressive cerebrospinal fluid (CSF) microenvironment. Border-associated macrophages originating from the dura mater can migrate into the CSF, and upregulated SPP1 enhances MMP14 expression, thereby promoting macrophage migration. Once in the CSF, these macrophages suppress the antitumor activity of CD8^+^ T cells and support leptomeningeal tumor progression. Targeting the SPP1-MMP14 axis partially reverses these changes [102], suggesting that this pathway may represent a functionally supported macrophage-related mechanism in LM.

This pattern is further supported by studies of CNS metastasis in NSCLC. Pial metastases are enriched in SPP1-positive macrophages, whereas parenchymal brain metastases retain more CXCL9-positive macrophage and lymphocyte-associated immune features. In LM, SPP1-positive macrophages are associated with impaired antigen presentation and upregulation of immunosuppressive genes [103]. These findings suggest that SPP1/OPN is more strongly linked to macrophage accumulation, CSF-compartment immunosuppression, and leptomeningeal metastatic progression than to parenchymal brain metastasis. Thus, in lung cancer-associated CNS metastases, SPP1/OPN should be interpreted as a site-specific macrophage-associated signal, with stronger evidence in leptomeningeal and pial metastatic niches than in parenchymal brain metastases.

## 7. Prospects for Translation and Methodological Challenges

OPN has potential value as a biomarker and therapeutic target, but its clinical translation remains challenging. OPN levels may increase in various CNS-related disorders and may reflect inflammation, tissue remodeling, barrier disruption, vascular injury, or tumor-associated immune remodeling. However, OPN is produced by multiple CNS and non-CNS sources, and its biological effects are shaped by cell type, molecular form, receptor usage, anatomical compartment, and disease stage. Therefore, OPN should not be interpreted as a uniformly harmful or beneficial molecule, nor should circulating OPN be assumed to reflect CNS-derived OPN without supporting compartment-specific evidence.

### 7.1. The Potential of OPN as a Biomarker

OPN can be detected in brain tissue, CSF, plasma, serum, and urine, and its levels have been associated with disease activity or pathological burden in several neurological disorders [104,105]. Evidence from CSF studies supports the relevance of secreted OPN in CNS diseases. In MS, CSF OPN has been reported to increase across the clinical spectrum and to show higher levels in active disease than in stable disease in some cohorts [15,106]. In AD-related studies, CSF OPN has been associated with synaptic dysfunction, tau pathology, neuronal injury, and immune activation [16]. In severe subarachnoid hemorrhage, longitudinal CSF and plasma measurements suggest a compartment-specific OPN response after acute vascular injury [17]. In CNS tumors, CSF OPN has been investigated as a diagnostic biomarker for CNS lymphoma and has also been reported in glioma-related studies, including evidence that GBM cells may secrete OPN into the CSF [18,24]. These findings support the inclusion of brain and CSF OPN in biomarker studies, but they also indicate that CSF OPN reflects a mixture of local CNS production, barrier status, inflammatory cell infiltration, and tumor-related secretion.

From a clinical perspective, OPN is more suitable as part of a state-sensing biomarker panel than as a standalone diagnostic marker. This is particularly important because OPN is not CNS-specific. In addition to resident CNS cells and CNS-associated compartments, OPN can be produced by peripheral immune cells, bone-related tissues, vascular cells, renal and urinary tract compartments, and tumor-associated tissues [107,108]. Therefore, plasma or serum OPN should be interpreted as a systemic adjunctive marker that may be influenced by peripheral immune activation, bone remodeling, vascular injury, renal function, tumor burden, and other non-CNS sources. CSF OPN is anatomically closer to the CNS compartment but can still be affected by blood–brain barrier disruption and systemic inflammation. Urinary OPN is more closely related to renal or urinary tract production, filtration, urinary proteolysis, urolithiasis-related mineral handling, and local urinary inflammation, and should not be considered a direct substitute for CSF or lesion-associated OPN in CNS diseases.

The molecular form being measured is another major limitation. Most CNS biomarker studies measure total OPN protein in CSF, serum, or plasma, or SPP1/Spp1 transcript abundance in tissue or single-cell datasets. These approaches usually do not distinguish full-length OPN, thrombin-cleaved OPN, MMP-generated fragments, intracellular OPN, or specific splice variants [10,13]. Therefore, current biomarker evidence should be interpreted as form-limited evidence of OPN-associated disease activity rather than proof that a defined OPN species mediates a specific pathological mechanism. Representative CNS-related biomarker contexts are summarized in Table 1.

Reported OPN concentrations also vary substantially across CNS-related conditions and biological compartments. In MS, meta-analytic evidence indicates that OPN levels are generally increased in CSF and peripheral blood, and that active MS is associated with higher CSF OPN levels than stable disease [45,109]. For MS, recent meta-analytic evidence also supports potential diagnostic and treatment-response relevance, including in the context of natalizumab response, while emphasizing assay and cohort heterogeneity [45]. In AD-related cohorts, CSF OPN has been reported to be elevated in AD or progressive MCI compared with neurological controls [110]. In severe subarachnoid hemorrhage, CSF OPN concentrations can exceed plasma concentrations, supporting a compartment-specific response after acute vascular injury [17]. In CNS malignancies, reported CSF OPN values differ across lymphoma, inflammatory CNS disease, MS, and GBM [18,24]. Because these values are influenced by assay platform, antibody specificity, sample matrix, BBB integrity, disease stage, treatment status, and whether total or fragment-specific OPN is measured, OPN concentrations should be interpreted relative to matched controls within the same study rather than as universal cross-disease thresholds.

Sex may also influence OPN biomarker interpretation. Although sex-related differences in circulating OPN have been reported in selected systemic inflammatory or disease contexts, current CNS and neuroinfectious datasets remain insufficient to define a consistent male- or female-specific OPN response pattern. In MS-related CSF studies, OPN levels appear to be more closely associated with disease activity or inflammatory CNS involvement than with patient sex [15]. Future biomarker studies should report sex distribution, adjust for sex when appropriate, and perform sex-stratified analyses when sample size permits.

Overall, biomarker interpretation should distinguish lesion-associated cellular OPN, CSF OPN, systemic circulating OPN, and urinary OPN. CSF OPN is anatomically closer to CNS pathology but remains influenced by barrier disruption and systemic inflammation, whereas plasma, serum, and urine provide less CNS-specific information. Therefore, circulating or urinary OPN should be used as adjunctive markers unless supported by matched CSF, imaging, tissue, or cell-source evidence.

### 7.2. The Potential of OPN as a Therapeutic Target

OPN-related pathways may provide therapeutic entry points in CNS diseases, but therapeutic design should account for disease stage, cellular source, receptor axis, and lesion compartment. OPN has been implicated in inflammatory amplification, remyelination failure, immunosuppression, and pathological remodeling in selected contexts, while it may also contribute to debris clearance, barrier repair, angiogenesis, and adaptive tissue remodeling after acute injury [28,33,35]. Therefore, the therapeutic question is not simply whether OPN should be reduced, but whether a given OPN-related pathway is sustaining pathology or supporting repair in a specific disease stage and lesion compartment. Premature or excessive inhibition during acute injury could interfere with endogenous repair, whereas selective modulation may be beneficial when sustained OPN signaling contributes to chronic inflammatory lesions, demyelinating environments, glial scars, or tumor-associated myeloid niches.

Accordingly, OPN-directed therapeutic strategies should be framed as stage-specific, cell-type-specific, and receptor-axis-specific interventions rather than broad systemic OPN blockade. Potential strategies may include modulation of disease-relevant SPP1/Spp1-expressing cell states, blockade of defined receptor pathways such as OPN-CD44 or OPN-integrin signaling, or inhibition of specific tumor-associated modules such as OPN-ITGA5 signaling linked to T-cell suppression and anti-PD-1 resistance [8,31,100]. This caution is particularly important in neuro-oncology. Although SPP1/Spp1-positive macrophages, OPN-CD44 signaling, and OPN-ITGA5-associated immune suppression provide plausible therapeutic entry points, they should not be interpreted as evidence that systemic OPN inhibition is ready for clinical application. Future therapeutic studies should define the OPN-producing cell population, dominant receptor pathway, disease stage, local tissue compartment, and potential effects on vascular repair, immune surveillance, and tissue homeostasis before advancing OPN-directed interventions.

### 7.3. The Application of New Technologies and Methods

To improve cross-disease readability, Table 2 summarizes the major CNS-related contexts according to disease context, OPN-associated cellular sources, receptor axes, model or human material, proposed functions, and evidence strength.

Much of the controversy surrounding OPN arises from methodological limitations in detecting SPP1/Spp1 transcripts and OPN protein across platforms. Single-cell RNA sequencing may underestimate Spp1 expression because of low transcript capture efficiency, dropout events, limited sequencing depth, or dissociation-related loss or alteration of activated macrophage and microglial states [111,112,113]. Single-nucleus RNA sequencing captures nuclear transcripts rather than the full cytoplasmic mRNA pool and may therefore differ from whole-cell transcriptomic profiles [114]. In addition, SPP1/Spp1 encodes a secreted protein, and extracellular OPN can persist, diffuse, or accumulate in tissue and CSF even when transcript abundance is low in sampled cells [18,24]. These issues may lead to discordance between SPP1/Spp1 transcript abundance, OPN protein localization, and measured OPN concentrations in CSF or plasma.

Protein-level and model-related limitations also affect interpretation. Antibody-based detection of OPN may be influenced by epitope recognition, post-translational modification, proteolytic cleavage, and species reactivity, making it difficult to compare immunostaining, Western blotting, ELISA, and spatial protein assays [115]. Mouse glioma and injury models do not fully reproduce the cellular heterogeneity, immune architecture, vascular niche, treatment history, or chronic disease evolution of human CNS disorders [116,117]. Moreover, secreted OPN can diffuse through the extracellular matrix, CSF, or circulation, so its detected location does not necessarily identify the producing cell type. Thus, OPN localization, CSF concentration, or SPP1/Spp1 expression alone is insufficient to define cellular origin or functional causality.

A stepwise validation framework may help reduce overinterpretation of OPN-related findings. Single-cell or single-nucleus transcriptomics can identify SPP1/Spp1-expressing cell populations, whereas spatial transcriptomics or RNAscope can determine whether these populations are located in lesion-relevant compartments, such as demyelinating lesion borders, infarct cores, perivascular spaces, glial scars, CSF-contacting regions, or tumor-associated myeloid niches. Protein-level assays, including immunostaining, spatial proteomics, ELISA, or mass spectrometry, are then needed to verify local or secreted OPN. Functional causality should then be tested using genetic, antibody-based, receptor-specific, pharmacological, and rescue approaches, ideally with defined OPN forms or receptor axes when available [7,28,100]. Time-series designs are also needed to distinguish transient repair-associated OPN induction from sustained OPN signaling in chronic inflammation, remyelination failure, scar remodeling, or tumor immunosuppression.

Finally, expression-based and inference-based approaches should be distinguished from mechanistic evidence. scRNA-seq, snRNA-seq, spatial transcriptomics, colocalization, and ligand–receptor inference can identify candidate OPN-producing cell states or potential OPN-CD44, OPN-integrin, or OPN-ITGA5 communication axes, but they do not prove that OPN directly mediates the observed biological outcome. By contrast, mechanistic evidence depends on functional perturbation and rescue strategies that directly test whether OPN or a defined receptor axis mediates the observed biological effect. This distinction is particularly important for Spp1-positive microglial or macrophage states described in ischemic stroke, temporal lobe epilepsy, Alzheimer’s disease, glioblastoma, and leptomeningeal metastasis.

## 8. Summary and Outlook

Across CNS development, acute injury, chronic neurological disease, and neuro-oncology, OPN is best viewed as a context-sensitive remodeling signal rather than a uniformly protective or pathogenic molecule. During development, OPN is associated with tissue boundary maintenance, white matter maturation, microglial remodeling, and neural circuit specialization. After acute CNS injury, OPN may participate in debris clearance, barrier repair, vascular remodeling, angiogenesis, and selected regenerative responses. In chronic neurological diseases, OPN is associated with demyelination, gliosis, synaptic dysfunction, vascular injury, and disease-associated myeloid states. In neuro-oncology, OPN-related signaling contributes to tumor–myeloid interactions, immunosuppression, vascular adaptation, tumor-cell plasticity, and therapy resistance. These diverse roles indicate that OPN should not be interpreted as a uniformly protective or pathogenic molecule, but rather as a molecular signal whose function depends on cellular source, receptor axis, molecular form, lesion compartment, and disease stage.

Despite increasing evidence, several major questions remain unresolved. It is still unclear which OPN-producing cell populations are functionally relevant in each disease context, because SPP1/Spp1 expression may arise from resident microglia, infiltrating macrophages, astrocytes, neurons, endothelial or perivascular cells, tumor cells, or tumor-associated macrophages. The receptor pathways that mediate disease-specific OPN effects also remain incompletely defined. CD44-, integrin-, and ITGA5-related signaling have been implicated in different settings, but many studies still rely on ligand–receptor inference rather than receptor-specific perturbation. Another key issue is the temporal boundary between reparative and pathological OPN activity. OPN may support debris clearance, barrier repair, angiogenesis, and adaptive remodeling after acute injury, whereas persistent OPN signaling may stabilize chronic inflammation, remyelination failure, glial scarring, tumor immune suppression, or therapy resistance. In addition, the distinct roles of extracellular and intracellular OPN in CNS diseases remain poorly understood, particularly because transcriptomic detection of SPP1/Spp1 does not define protein localization, secretion, or intracellular function.

Future studies should therefore move beyond disease-associated expression patterns and integrate spatial localization, protein-level validation, time-series analysis, and functional perturbation. Cell-source-specific models, receptor-axis perturbation, form-defined OPN assays, and rescue experiments will be needed to determine whether OPN acts as a local effector, a fluid biomarker, a marker of disease-associated cell states, or a functionally relevant therapeutic node. For translational development, the central challenge is to determine whether OPN-related pathways can be targeted without disrupting beneficial repair, vascular remodeling, immune surveillance, or tissue homeostasis. Addressing these questions will be essential for developing mechanism-based OPN biomarkers and stage-, cell type-, or receptor-axis-specific intervention strategies.

## Figures and Tables

**Table 1:** Biomarker interpretation of OPN in CNS-related conditions.

Disease	Sample	Biomarker Interpretation
MS	CSF, serum/plasma, lesion tissue	CNS inflammation, lesion activity, disability progression
AD	CSF, brain tissue, single-cell/spatial datasets	Plaque immune remodeling, synaptic injury, amyloid response
AE	CSF, serum	Active CNS immunopathology and inflammatory severity
Stroke/SAH	Brain tissue, CSF, plasma/serum	Acute injury, BBB disruption, vascular remodeling
TBI/SCI	Injured tissue, CSF, experimental datasets	Glial activation, scar remodeling, vascular repair
CNS tumors	Tumor tissue, CSF, single-cell/spatial datasets	TAM remodeling, immunosuppression, angiogenesis, therapy resistance
Neuroinfections	Brain; CSF; plasma/serum	Compartment-specific inflammatory signal

Abbreviations: AD, Alzheimer’s disease; AE, autoimmune encephalitis; BBB, blood–brain barrier; CNS, central nervous system; CSF, cerebrospinal fluid; MS, multiple sclerosis; OPN, osteopontin; SAH, subarachnoid hemorrhage; SCI, spinal cord injury; TAM, tumor-associated macrophage; TBI, traumatic brain injury.

**Table 2:** OPN-associated cellular sources, receptor axes, functions, and evidence strength across CNS-related contexts.

Disease	Cellular Sources	Receptor Axes	Model/Material	Proposed Functions	Evidence Strength
CNS development	Microglia; CD11c^+^ microglia; neurons	Integrins; ERK/PI3K-AKT	In vivo; human	Tissue protection; myelination; circuits	Mixed functional/correlative evidence
MS/demyelination	Astrocytes; glia; immune cells	OPN-CD44; integrins	In vivo; human	Inflammation; remyelination failure	Mixed functional/correlative evidence
TLE	Reactive microglia; astrocytes; OPCs	OPN-CD44	Human	Hippocampal inflammation; glial crosstalk	Mainly correlative/omics evidence
ALS	Motor neurons; extracellular OPN sources	αvβ3/MMP-9; CD44	In vivo; human	Astrocyte migration; microglial phagocytosis	Mixed functional/correlative evidence
AD/cognitive disorders	Plaque microglia; perivascular macrophages; fibroblasts	CD44; integrins	In vivo; in vitro; human	Plaque remodeling; amyloid response; synaptic loss	Mixed functional/correlative evidence
Stroke	Activated microglia; myeloid cells	OPN-CD44	In vivo; human	Debris clearance; glial recruitment; inflammation	Mixed functional/correlative evidence
ICH/SAH	Macrophages; vascular cells	CD44/P-gp; Nrf2/HO-1; BDNF	In vivo; human	BBB repair; vascular protection	Selected functional evidence
TBI	Microglia; macrophages; reactive glia	MMP9-CD44	In vivo	Debris clearance; synaptic remodeling; scarring	Mixed functional/correlative evidence
SCI	Microglia; macrophages; endothelial cells	Spp1-angiogenic; mTOR	In vivo	Angiogenesis; axon remodeling	Mixed functional/correlative evidence
GBM	Tumor cells; macrophages; TAMs	OPN-CD44; integrins; ITGA5	In vivo; in vitro; human	TAM remodeling; angiogenesis; stemness	Mixed functional/correlative evidence
GBM resistance	SPP1/Spp1^+^ macrophages	OPN-ITGA5	In vivo; human	T-cell suppression; anti-PD-1 resistance	Selected functional evidence
LM/CNS metastasis	Border macrophages; SPP1/Spp1^+^ macrophages	SPP1/Spp1-MMP14; OPN-CD44/PTGER4	In vivo; human	CSF migration; immune suppression	Mixed functional/correlative evidence
Neuroinfections	Microglia; macrophages; neurons	OPN–integrins; OPN–CD44	In vivo; human	Neuroinflammation; host response	Mixed functional/correlative evidence

Abbreviations: AD, Alzheimer’s disease; ALS, amyotrophic lateral sclerosis; BBB, blood–brain barrier; CNS, central nervous system; CSF, cerebrospinal fluid; GBM, glioblastoma; ICH, intracerebral hemorrhage; LM, leptomeningeal metastasis; MS, multiple sclerosis; OPN, osteopontin; OPCs, oligodendrocyte precursor cells; SAH, subarachnoid hemorrhage; SCI, spinal cord injury; TAMs, tumor-associated macrophages; TBI, traumatic brain injury; TLE, temporal lobe epilepsy.

## Data Availability

No new data were created or analyzed in this study. Data sharing is not applicable.
